# Dynamic Duo—The Salmonella Cytolethal Distending Toxin Combines ADP-Ribosyltransferase and Nuclease Activities in a Novel Form of the Cytolethal Distending Toxin

**DOI:** 10.3390/toxins8050121

**Published:** 2016-04-25

**Authors:** Rachel Miller, Martin Wiedmann

**Affiliations:** Department of Food Science, Cornell University, Ithaca, NY 14850 USA; ram524@cornell.edu

**Keywords:** cytolethal distending toxin, *Salmonella*, nontyphoidal, typhoid toxin, DNA damage, virulence factor

## Abstract

The cytolethal distending toxin (CDT) is a well characterized bacterial genotoxin encoded by several Gram-negative bacteria, including *Salmonella enterica* (*S. enterica*). The CDT produced by *Salmonella* (S-CDT) differs from the CDT produced by other bacteria, as it utilizes subunits with homology to the pertussis and subtilase toxins, in place of the traditional CdtA and CdtC subunits. Previously, S-CDT was thought to be a unique virulence factor of *S. enterica* subspecies *enterica* serotype Typhi, lending to its classification as the “typhoid toxin.” Recently, this important virulence factor has been identified and characterized in multiple nontyphoidal *Salmonella* (NTS) serotypes as well. The significance of S-CDT in salmonellosis with regards to the: (i) distribution of S-CDT encoding genes among NTS serotypes, (ii) contributions to pathogenicity, (iii) regulation of S-CDT expression, and (iv) the public health implication of S-CDT as it relates to disease severity, are reviewed here.

## 1. Introduction

*Salmonella enterica* (*S. enterica*) is a Gram-negative bacterium that causes gastrointestinal illness in humans and animals. The genus *Salmonella* includes two species, *enterica* and *bongori*. Within the species *S. enterica*, there are six subspecies: *enterica* (subspecies I), *salamae* (subspecies II), *arizonae* (subspecies IIIa), *diarizonae* (subspecies IIIb), *houtenae* (subspecies IV), and *indica* (subspecies VI) [1]. Subspecies *enterica* is further categorized into 1586 serotypes (e.g., Typhimurium, Typhi, Newport, and Enteritidis), representing unique antigenic formulae of the O and H antigens [2]. For simplicity, *S. enterica* serotypes may be further categorized as “typhoidal” (*i.e.*, *S. enterica* serotype Typhi [*S.* Typhi]), “paratyphoidal” (*i.e.*, *S. enterica* serotypes Paratyphi A, B, or C) or nontyphoidal (*i.e.*, *S. enterica* serotypes except Typhi, and Paratyphi A, B, or C) [3].

Salmonellosis, the disease resulting from a *Salmonella* infection, is primarily acquired through the consumption of contaminated food or water. In the US, foodborne salmonellosis accounts for an estimated 1.03 million cases of foodborne illness per year [4]. Internationally, nontyphoidal salmonellosis is responsible for an estimated 80.3 million illnesses and 150,000 deaths per year [5]. Importantly, some serotypes (e.g., Typhimurium, Newport, and Enteritidis) are capable of causing disease in a wide range of hosts, including humans and other mammals, birds, and reptiles, while others are host-restricted (e.g., *S*. Typhi in humans) [6,7].

Although not fully understood, *S. enterica* serotypes differ in virulence, with some serotypes being more commonly associated with invasive disease, and others causing a self-limiting gastroenteritis [6]. *S.* Typhi, the causative agent of typhoid fever, causes a severe, sometimes life-threatening illness. Serotypes Paratyphi A, B, and C cause a similar illness known as paratyphoid fever [3]. Whole genome sequence comparisons of serotype Typhi and nontyphoidal serotypes have failed to definitively account for differences in virulence [8,9]. Recently, *S.* Typhi was found to encode a variant of the cytolethal distending toxin (CDT), an important virulence factor for several other Gram-negative bacteria [10]. This novel form of CDT (hereafter referred to as “S-CDT” for Salmonella CDT) was believed to be unique to *S.* Typhi, leading to its classification as the “typhoid toxin” [8,11,12]. However, S-CDT has since been identified in at least 40 NTS serotypes [13]. Our current understanding of S-CDT with regards to its regulation, structure, function, and mechanism of action has primarily been informed by characterization of S-CDT produced by *S.* Typhi. The established genetic and pathogenic differences among *S. enterica* serotypes, particularly Typhi and nontyphoidal serotypes, warrant further characterization of S-CDT among different NTS serotypes. This review will: (i) summarize the current understanding of the distribution, production, structure and function, and cytotoxic effects of S-CDT produced by *S. enterica* serotypes; and (ii) compare the unique features of S-CDT to the CDTs produced by other Gram-negative bacteria.

## 2. *Salmonella* Encodes a Novel Form of CDT

CDT was first characterized in *Escherichia coli* (*E. coli*) in the late 1980s by Johnson and Lior, who noted that eukaryotic cells which were co-incubated with filtrates of overnight cultures of *E. coli* appeared distended, and arrested in the G2/M phase [14]. Subsequent analyses also identified CDT production by other Gram-negative pathogens, including *Campylobacter* spp. [15,16,17,18], *Haemophilus* spp. [19,20], *Aggregatibacter actinomycetemcomitans* (*A. actinomycetemcomitans*) [21], *Helicobacter* spp. [22,23,24,25,26], *Shigella* spp. [27], *Yersinia* spp. [10], *Providencia alcalifaciens* [28], and *S.* Typhi [12,29]. The CDT encoded by all of these pathogens, with the exception of *S.* Typhi, exists as a tripartite AB_2_ toxin encoded by the genes *cdtA, cdtB,* and *cdtC*, with the CdtB subunit serving as the active component of the toxin, and subunits CdtA and CdtC implicated in binding to host cells and subsequent intracellular trafficking [10]. S-CDT represents an important exception, as *Salmonella* strains producing S-CDT encode *cdtB*, but not *cdtA* or *cdtC* [11,12,29]. In contrast to the AB_2_ configuration of CDTs encoded by other Gram-negative bacteria, S-CDT is an A_2_B_5_ toxin, comprised of toxin subunits: (i) CdtB (encoded by *cdtB*, cytolethal distending toxin subunit B), a nuclease subunit; (ii) PltA (encoded by *pltA*, pertussis like toxin subunit A), an ADP-ribosylating toxin subunit; and (iii) PltB (encoded by *pltB*, pertussis like toxin subunit B), serving as a pentameric ring constituting the binding subunit [11]. Subunits PltA and PltB share homology with the Ptx S1 (active) and Ptx S2 (binding) subunit, respectively, of the pertussis toxin, which ADP-ribosylates host G proteins [11,30]. Similarly, the 3D configuration of the PltB subunit also aligns well with the binding subunit (SubB) of the *E. coli* subtilase toxin, which is a serine protease [8,31]. Recent studies have identified genes encoding S-CDT in a number of NTS serotypes as well [9,13,32,33,34]. To date, genes encoding S-CDT (*i.e.*, genes *pltA*, *pltB*, and *cdtB*) have been characterized in at least 40 NTS serotypes (see Table 1) [9,13,32]. Amino acid alignments of CdtB, PltA, and PltB from both NTS serotypes and serotype Typhi suggest that these proteins are highly conserved among *S. enterica* serotypes [34]. Genomic analyses have also detected orthologs of genes encoding S-CDT in *S. bongori* and *S. enterica* subsp. *arizonae*, although the functionality of these gene products has not been assessed [34,35]. Further DNA-based analyses will aid in the characterization and detection of genes encoding S-CDT in other NTS serotypes, and will likely expand the list of NTS serotypes known to encode S-CDT.

## 3. Regulation of S-CDT Expression

Several reports have confirmed that S-CDT expression is restricted to intracellular *S.* Typhi residing within the salmonella containing vacuole (SCV) [11,12]. Importantly, this is in contrast to CDT production by other Gram-negative bacteria, for which the toxin is routinely detected in cell-free supernatants of CDT positive strains cultivated in standard laboratory media [14,19,37,38]. The intracellular requirement for S-CDT production has not yet been confirmed for NTS expressing CDT.

The requirement of bacterial internalization for S-CDT expression by *S.* Typhi has been confirmed at both the transcriptional and translational levels. Haghjoo and Galán used a luciferase reporter strain to establish that *cdtB* is not expressed by *S.* Typhi grown in lysogeny broth (LB), and that transcription was only activated when *S.* Typhi was allowed to infect eukaryotic cells [12]. Furthermore, epithelial cells infected with an invasion-deficient mutant of *S.* Typhi did not have the characteristic distended phenotype, nor did they arrest in the G2/M phase, suggesting that invasion, and not just adhesion, is required for S-CDT production by *S.* Typhi [12]. However, transcription of *pltA* and *pltB* can be detected when *S.* Typhi is grown in standard LB media, although at very low quantities [11]. This is likely due to the organization of the CdtB-islet into two distinct operons encoding the toxin subunits [11]. Taken together, the fact that *pltA* and *pltB* are located in an operon separate from *cdtB*, and that transcription of *pltA* and *pltB,* but not *cdtB,* may occur in standard culturing medium, suggests that *pltA* and *pltB* may be regulated separately of *cdtB*.

A transposon mutagenesis screen identified IgeR, a transcriptional regulator belonging to the DeoR family of transcriptional regulators, as a repressor of *cdtB* transcription in *S.* Typhi [39]. *In vitro* analyses determined that IgeR is able to bind to the *cdtB* promoter, and effectively suppress *cdtB* expression [39]. Likewise, deletion of *igeR* was sufficient to de-repress *cdtB* expression in LB media, a normally non-permissive environment for *cdtB* expression by *S.* Typhi [39]. IgeR also plays a role in the regulation of other genes involved in virulence, including SPI-1 encoded type three secretion system (TTSS) components, flagellar proteins, and SPI-1 TTSS effector proteins, as deletion of *igeR* resulted in decreased expression of these genes [39]. IgeR is conserved among *S. enterica* subsp. *enterica* serotypes, and hence could also control transcription for S-CDT production in NTS [39]. In addition, *cdtB* transcription was found to be activated concurrently with *parE* and *mntR*, but repressed with transcription of *potG* and *tldD,* although the exact mechanisms regarding their regulation are currently unknown [39]. In support of IgeR-mediated repression of *cdtB* transcription, plasmid-based expression of *cdtB* under control of its native promoter, in a heterologous bacterial host (*i.e.*, *S.* Typhimurium), was found to be sufficient for constitutive expression of the CdtB-islet under conditions that are normally non-permissive for wild type strains of *S.* Typhi [39,40]. Another study suggested that the two component PhoQ-PhoP regulatory system may also play a role in *cdtB* expression in *S.* Typhi, as increased levels of *cdtB* mRNA transcripts and CdtB were detected when *Salmonella* cells were subjected to PhoP-inducing conditions [41]. As the CdtB-islet constitutes two operons, and expression of the *pltAB* operon may be detected when S-CDT positive strains are cultured under conditions that are normally non-permissive for expression of the operon containing *cdtB*, it is unclear whether IgeR also regulates transcription of the *pltAB* operon. Taken together, these results suggest that regulation of S-CDT expression in *S.* Typhi at the transcriptional level involves multiple regulatory components, which are likely also involved in the regulation of invasion-associated genes.

**Figure 1:**
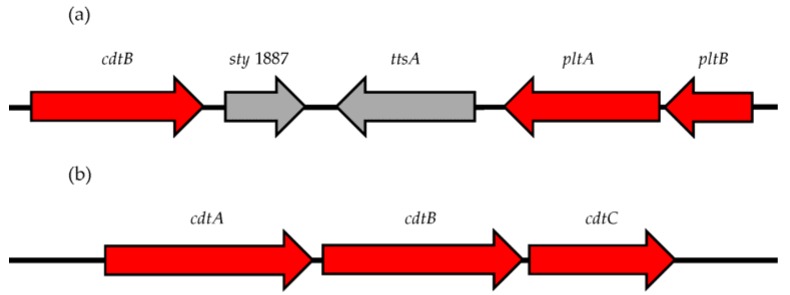
Comparison of the CdtB-islet encoded by: (**a**) *Salmonella enterica* serotypes; and (**b**) all other Gram-negative species producing a cytolethal distending toxin (CDT). (**a**) The *Salmonella* CDT (S-CDT) is comprised of subunits PltA, PltB, and CdtB encoded by *pltA, pltB*, and *cdtB*, respectively. The CdtB-islet in *Salmonella* also encodes two genes (*sty1887* and *ttsA*) which are implicated in toxin secretion but are not subunits of S-CDT [39,42]. (**b**) Genes *cdtA, cdtB,* and *cdtC* encode the CDT for *Aggregatibacter actinomycetemcomitans*, *Campylobacter* spp., *Escherichia coli, Haemophilus* spp., *Helicobacter* spp., *Providencia alcalifaciens*, *Shigella* spp., and *Yersinia* spp. Genes colored red compose the CDT and S-CDT; genes shown in gray are present in the CdtB-islet of *Salmonella*, but do not encode subunits of the S-CDT.

Two additional genes, *sty1887* and *sty1889* within the CdtB-islet, are implicated in S-CDT gene regulation as well (see Figure 1) [42]. Deletion of *sty1889* (renamed *ttsA*), but not *sty1887*, abrogated secretion of S-CDT in a *S.* Typhi strain, and prevented subsequent intoxication of epithelial cells [42]. *In silico* analysis implicates that *ttsA* encodes a *N*-acetyl-β-d-muramidase, with homology to a bacteriophage muramidase [42]. Similar to CdtB, TtsA is not detected in standard LB culturing medium, and is only detected when *S.* Typhi infects a host cell [42]. Further analyses determined that the TtsA peptidoglycan binding domain is required for S-CDT secretion [42]. Currently, all S-CDT regulatory analyses have been performed in serotype Typhi. Due to the marked differences between Typhi and NTS serotypes, it will be important to characterize the regulation and expression of S-CDT in NTS.

## 4. ArtA and ArtB and Their Relationship to S-CDT

Homologs to genes encoding the PltA and PltB subunits of S-CDT have also been detected in a number of NTS serotypes [13]. First identified in *S. enterica* serotype Typhimurium strain DT104, genes encoding an ADP-ribosyl transferase toxin homolog (*artA* and *artB*) have been characterized on a putative prophage in serotype Typhimurium, as well as in other NTS serotypes [13,43]. The protein encoded by *artA* is homologous to both the pertussis-like toxin subunit in *S.* Typhi (encoded by *pltA*) and the S1 subunit of the pertussis toxin (encoded by *ptxA*), with the predicted amino acid products sharing 59% and 33% amino acid identity, respectively [43]. A second subunit, ArtB, has homology to the amino acid product encoded by *pltB* (30% amino acid identity), as well as the S2 and S3 subunits (30.7% amino acid identity) of the Ptx binding component of the pertussis toxin [43]. Genome alignments have detected *artA* and *artB* in the majority of NTS serotypes encoding the CdtB-islet [13]. For these serotypes, the location of *artA* and *artB* was inconsistent, providing support for the genes being encoded on a prophage [13]. Despite the seemingly widespread distribution of *artA* and *artB*, the function and potential contributions of *artA* and *artB* gene products to virulence remain unknown. Likewise, it is unclear if *artA* and *artB* are expressed concurrently with genes in the CdtB-islet. While all three subunits of S-CDT are required for full activity, some studies have shown that deletion mutants of *pltB* retain some residual cytotoxic activity [32]. Therefore, it would be interesting to examine whether ArtA or ArtB, or both, can potentially substitute for PltA or PltB. A recent study analyzing the 3D crystal structure of S-CDT from *S.* Typhi predicted that three cysteine residues in the PltA subunit serve as the physical link between CdtB and PltA [8]. In contrast, the ArtA subunit only contains two such cysteine residues that could interact with CdtB, and therefore CdtB is predicted to be preferentially bound by PltA rather than ArtA [8]. ArtA and ArtB appear to be more widespread among NTS, as they are also present in strains that do not encode S-CDT [43]. However, the activity and effects on host cellular processes resulting from the “ArtAB toxin” remain uncharacterized.

## 5. Structure and Function of S-CDT

**Figure 2:**
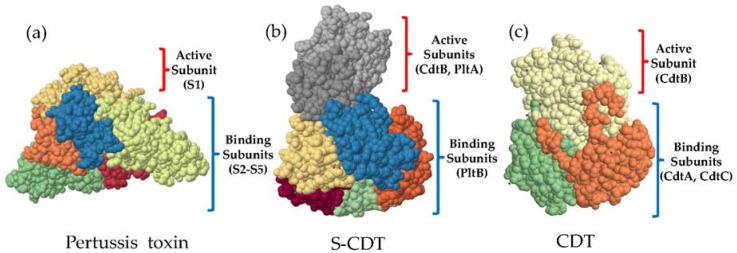
Space-fill models of the 3D structure of the: (**a**) pertussis toxin; (**b**) cytolethal distending toxin (CDT) from *Salmonella enterica* serotype Typhi; and (**c**) CDT from *Haemophilus ducreyi*. The unique A_2_B_5_ structure of the Salmonella cytolethal distending toxin (S-CDT) combines active subunits CdtB from the CDT produced by other Gram-negative species, and the ADP-ribosyltransferase toxin subunit of the pertussis toxin. The binding subunit of S-CDT is arranged as a pentameric ring, similar to the binding portion of the pertussis toxin. Protein databank accession numbers: Pertussis toxin (1PRT) [46], S-CDT (4K6L) [8], and CDT (1SR4) [47].

S-CDT is arranged in an A_2_B_5_ configuration (see Figure 2) [8]. In its final quaternary form the toxin exists as a pyramid-shaped structure that is ~90 Å tall with a maximum width of ~60 Å (at the base) [8]. Five PltB subunits (encoded by a single copy of *pltB*) form a pentameric ring at the base of the toxin [8]. The pentameric ring is covalently linked to PltA at its carboxy terminus, which inserts into the hydrophobic alpha-helical ring of the PltB pentamer [8]. A disulfide linkage between PltA Cys214 and CdtB Cys269 anchors CdtB at the most distal location from S-CDT’s pentameric base (Figure 2) [8]. Therefore, CdtB does not physically interact with the PltB subunits [8]. Structurally, the disulfide bonds and catalytic residues of both the pertussis toxin S1 (Glu129) and the PltA subunit of S-CDT (Glu 133) overlap in the 3D configuration, suggesting that reduction of the disulfide bonds would be necessary for activation of the ADP-ribosylating function of PltA, as is the case for the pertussis toxin [8]. Alignment of the 3D protein structures of the S-CDT subunits PltA, PltB, and CdtB with their respective homologous protein subunits (*i.e.*, Ptx S1 with PltA, Ptx S2 or SubB with PltB, and CdtB from *S*. Typhi with CdtB from *Haemophilus ducreyi* (*H. ducreyi*)) yielded low root-mean-square-deviations [8]. This further supports the hypothesis that the subunits PltA and PltB share homology to subunits of the pertussis and subtilase toxins, respectively, and also share a common structure and function (Figure 2) [8].

The translated product of *pltB*, encoding the pentameric B-subunit of S-CDT, is 137 amino acids in length, composed of a 23 aa secretion signal peptide and a 114 aa chain [44]. Interestingly, the amino acid sequence of PltB, as well as the 3D configuration, aligns well with the SubB subunit of the subtilase toxin encoded by *E. coli* [8,31]. Analogous to SubB, PltB is implicated in binding to host cells [8,31]. Chromatography-based interaction studies have identified several possible host cell receptors for the PltB subunit of S-CDT, namely podocalyxin-like protein 1 (PODXL), but also a variety of sugar moieties on glycoproteins and glycoplipids, including sialylated glycans [8]. Given S-CDT’s ability to intoxicate a wide variety of cell types, it is likely that PltB is able to bind to a variety of host cell structures, namely glycans [8]. Similarly, the SubB subunit of the subtilase toxin preferentially recognizes and binds to sialylated glycoproteins [31]. There are conflicting reports regarding the requirement of PltB for cytotoxicity [11,32]. A Δ*pltB* mutant of *S.* Typhi failed to induce a G2/M cell cycle arrest in a cell culture model, suggesting that PltB plays a critical role in toxin trafficking [8,12]. However, HeLa cells infected with a Δ*pltB* mutant of *S. enterica* serotype Javiana (*S.* Javiana) showed evidence of a G2/M phase arrest, consistent with S-CDT [32]. Purified PltB has been shown to up-regulate chemokine and cytokine production in a cell culture model as well, suggesting that its role in virulence may not be limited to just ensuring delivery of CdtB to host cells [45].

PltA, one of S-CDT’s two active subunits, is a functional ADP-ribosylating subunit with homology to the active subunit of the pertussis toxin [8,11,13]. The 27.1 kDa PltA subunit consists of 242 aa, comprising both a signal sequence peptide of 18 aa residues and a 224 aa chain [48]. The functionality of PltA as an ADP-ribosyltransferase has been confirmed in *S*. Typhi, however the host protein target(s) remain(s) unknown [11]. In *Bordetella pertussis*, the causative agent of whooping cough, the pertussis toxin plays a critical role in modulating the host immune response by ADP-ribosylating host G proteins, and subsequently disrupting G protein signaling pathways [30,49,50]. Importantly, eukaryotic cells infected with *S.* Typhi Δ*pltA* mutants do not have the characteristic distended phenotype [11,32]. However, substitution of PltA with a catalytically inactive variant PltA^E133A^, restored S-CDT-induced cytotoxicity, suggesting that despite PltA’s functioning as an active ADP-ribosylating toxin, its role in S-CDT-mediated cytotoxicity is most likely related to entry and trafficking of S-CDT in intoxicated eukaryotic cells, as the subunits CdtB and PltB do not physically interact [8,11]. Further elucidation of the molecular targets of PltA-mediated ribosylation will be necessary to fully understand its role as a virulence factor, and furthermore, its role in S-CDT-mediated cytotoxicity. While PltA does not appear to play an important role in the DNA damaging activity of the CdtB subunit, it will be important to identify the molecular targets of the ADP-ribosyl transferase in order to elucidate its potential contributions to the outcome of an infection with a CDT positive strain.

The cytotoxic effects associated with S-CDT intoxication are primarily attributable to the CdtB subunit. The CdtB subunit has limited amino acid sequence homology to mammalian DNase I, and is thought to cleave host DNA, thereby triggering activation of the host cell’s DNA damage response (DDR), resulting in the distended morphology and G2/M cell cycle arrest [10,51]. The CdtB subunit may also act as a phosphatase, as the CDT produced by *A. actinomycetemcomitans* has demonstrated PI-3,4,5-triphosphate phosphatase activity, although phosphatase activity has yet to be confirmed for S-CDT [52]. The CdtB subunit is highly conserved among CDT positive *Salmonella* [34]. The CdtB subunit has a mass of 29.6 kDa, and is 269 aa in length, comprising a 22 aa signal peptide and 247 aa chain [48]. In agreement with characterization of the CDT produced by other Gram-negative bacteria, CdtB is necessary for the distended phenotype of infected cells, as deletion of *cdtB* in *S.* Typhi and NTS strains results in a loss of the ability to elicit a G2/M phase arrest in eukaryotic cells [11,12,32,34,51]. Transfection of a Cos-2 cell line with plasmid-encoded *S.* Typhi *cdtB* was sufficient for cytotoxicity, further supporting CdtB as the active component of S-CDT [12]. Despite CdtB’s confirmed activity, it is still unclear if CdtB preferentially targets certain DNA motifs, and how many single strand breaks (SSB) and/or double strand breaks (DSB) it may introduce into any given strand of DNA.

## 6. Mechanism of Action

The delivery and trafficking of S-CDT differs from that of the CDT produced by other Gram-negative bacteria. The key differences distinguishing S-CDT trafficking and activation from CDTs produced by other Gram-negative bacteria include: (i) S-CDT is only produced when *Salmonella* cells are residing within a host eukaryotic cell; (ii) S-CDT must be exported out of the SCV and subsequently out of the host cell, after which the exported S-CDT may either re-enter the cell or intoxicate a nearby cell; (iii) S-CDT’s unique A_2_B_5_ structure (compared to the AB_2_ configuration of other CDTs) requires a reducing atmosphere to dissociate the PltA and CdtB subunits; and (iv) the host cell receptors for S-CDT differ as a reflection of its use of PltB rather than CdtA and CdtC subunits for binding to host cells.

### 6.1. S-CDT Uses Multiple Host Cell Receptors Enabling it to Intoxicate a Wide Variety of Cell Types

Collectively, S-CDT and other CDTs are able to intoxicate a wide variety of host cells [8,51]. Despite this, several reports suggest that CDT binding and intracellular trafficking within host cells is species specific, with different receptors and intracellular trafficking mechanisms being utilized depending on the bacterial species producing the CDT [51,53,54,55,56]. A recent study suggested that S-CDT binds to a variety of host receptors, including PODXL, and CD45 on B and T cells [8]. Song *et al.* noted that sugar moieties of primarily glycoproteins, but also glycolipids, are the primary target for S-CDT binding [8]. S-CDT preferentially binds α(2-3)-linked *N-*acetylneuraminic acid [8]. In comparison, the B subunit of the subtilase toxin (which has homology to the B subunit of S-CDT) binds preferentially to α(2-3)-linked *N-*glycolylneuraminic acid terminating glycans, but also α(2-3)-linked *N-*acetylneuraminic acid glycans [31]. Similar to the pertussis toxin S2 binding subunit, S-CDT also demonstrates some affinity for terminal sialic acid moieties [8,30].

The CDTs produced by other Gram-negative bacteria may also use N-linked carbohydrate structures as receptors. Initially, *E. coli* CDT was characterized as binding to N-linked carbohydrate moieties of glycoproteins, while the CDT produced by *A. actinomycetemcomitans* preferentially uses the ganglioside GM3 as the cell receptor [57,58]. Eshraghi *et al.* noted that the CDTs produced by *E. coli*, *H. ducreyi, Campylobacter jejuni*, and *A. actinomycetemcomitans* were affected differently by host cell N-linked glycosylation, cholesterol levels, and deficiencies in sialic acid, galactose and glycolipids, therefore suggesting that the CDT mode of entry is dependent on the bacterial species producing the CDT [55]. In summary, like other CDTs, it appears that S-CDT does not utilize a single receptor. Rather, S-CDT can utilize multiple different receptors, perhaps explaining why S-CDT is capable of intoxicating a number of different cell types [8,34,40]. It has been suggested that the variability in host cell receptors utilized by CDTs from different bacterial species, may partially explain why certain CDT-producing pathogens preferentially inhabit and colonize particular regions of the host [10,51,55,59].

### 6.2. Entry and Trafficking of S-CDT

Intracellular trafficking and subsequent targeting of CdtB to the nucleus occurs via different mechanisms, depending on the bacterial species producing the CDT [10,56]. In the case of S-CDT, following its production by *Salmonella* residing within the SCV, it appears that S-CDT must first be exported out of the infected host cell, before being endocytosed by either the eukaryotic host cell from which it was produced, or by another cell [11]. The most convincing evidence for this hypothesis was generated by Spanò *et al.*, who showed that addition of a toxin-neutralizing antibody prevented intoxication of epithelial cells that were infected with *S.* Typhi cells actively producing S-CDT [11]. The secretion of S-CDT out of the SCV, and subsequently out of the host cell, requires the production of outer membrane vesicles (OMV), which “bud” off of the SCV, and are trafficked by host kinesin along microtubules to the plasma membrane [40].

**Figure 3:**
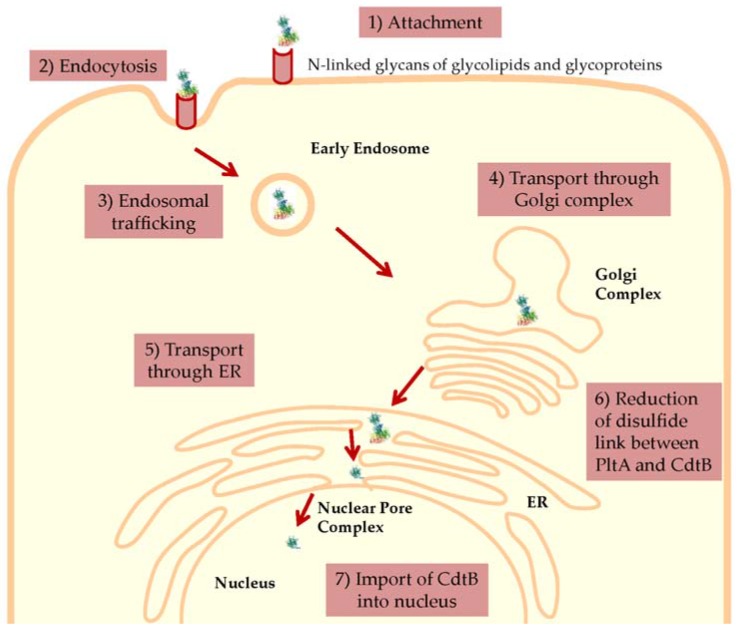
Proposed model for the entry and intracellular trafficking of Salmonella cytolethal distending toxin (S-CDT). (1) The PltB subunits of S-CDT bind to sugar moieties on glycoproteins and glycolipids on the host cell surface [8]. (2) S-CDT is internalized by endocytosis and is (3) trafficked in endosomes, which deliver the S-CDT to the Golgi complex [11]. Subsequently, S-CDT is (4) transported retrograde through the Golgi complex, likely mediated by COPI vesicles, and then (5) through the endoplasmic reticulum [53]. (6) It is hypothesized that in the ER, host reductases reduce the disulfide bonds covalently linking the PltA and CdtB subunits, releasing CdtB from the holotoxin [8]. (7) The CdtB subunit is imported into the nucleus, likely by passing through the nuclear pore complex, as is done for other CDTs [53]. Once in the nucleus, CdtB acts as a nuclease and cleaves host DNA to activate the host cell’s DNA damage response [40]. Protein databank entry for S-CDT (4K6L) [8]. Cell adapted from [63].

Re-entry of S-CDT into a eukaryotic cell infected with *Salmonella* (autocrine pathway), or entry into an uninfected cell (paracrine pathway), occurs via endocytosis (See Figure 3). Similarly, the pertussis toxin, subtilase toxin, and CDTs produced by other Gram-negative bacteria, also utilize endocytosis for toxin entry [30,31,51]. While the requirement of clathrin in the endocytosis of S-CDT is currently unknown, endocytosis of other CDTs may occur via clathrin-dependent or clathrin-independent mechanisms, while endocytosis of the subtilase toxin is clathrin-dependent [31,51,53,60]. Following endocytosis, S-CDT is predicted to follow retrograde trafficking through the Golgi complex and endoplasmic reticulum [40]. For *H. ducreyi*, endosomal trafficking transports the CdtB and CdtC subunits retrograde to the trans-Golgi network, and then subsequently through the Golgi complex via COPI vesicles, as evidenced by sulfation (a Golgi-specific activity) of CdtB, and the absence of a distended phenotype when intoxicated cells were treated with Brefeldin A, which inhibits the formation of COPI vesicles [59,61]. Subsequent transportation of S-CDT across the nuclear membrane, and into the nucleus of the host cell where it elicits SSB and/or DSB, is currently uncharacterized. It is still unclear how CdtB dissociates from the other components of S-CDT, and at which stage this occurs [8]. Presumably, the disulfide bond between PltA Cys214 and CdtB Cys269, is reduced by host cell reductases [8]. By comparison, the pertussis toxin active component is dissociated, and therefore activated, in the ER prior to being released into the cytosol where it ADP-ribosylates G proteins [30]. It is possible that S-CDT components PltA and CdtB separate in the ER as well. Following exit of the ER, the CdtB subunit must cross the nuclear membrane, and enter the host cell nucleus in order to induce DNA damage.

The current understanding of the intracellular trafficking of S-CDT is largely based on the intracellular trafficking of related toxins, namely the CDTs produced by other Gram-negative bacteria, and the pertussis and subtilase toxins. However, some studies have demonstrated that CDTs may utilize different intracellular trafficking mechanisms, requiring different components of the host cell for trafficking to the nucleus [56,62]. For example, treatment of HeLa cells with chemical agents blocking endosomal acidification (e.g., bafilomycin A1 or ammonium chloride) prevented *H. ducreyi* CdtB transportation to the nucleus, but not *E. coli* CdtB trafficking to the nucleus [56]. These results suggest that *E.coli* and *H. ducreyi* CDTs utilize different intracellular trafficking mechanisms to elicit their cytotoxic effects [56].

Importantly, S-CDT has multiple structural differences in comparison to the CDTs produced by other Gram-negative bacteria, namely, the absence of CdtA and CdtC subunits, and the presence of subunits PltA and PltB subunits. Therefore, further research will be necessary to confirm the exact trafficking mechanisms of S-CDT produced by Typhi and nontyphoidal serotypes.

## 7. S-CDT’s Role in Virulence

Characterizations of the deleterious effects associated with S-CDT intoxication at both the cellular and organismal levels have provided key insights into the contributions of S-CDT to disease.

### 7.1. DNA Damage and Induction of the DNA Damage Response

The hallmark of CDT-intoxication is the production of SSB and/or DSB, resulting in activation of the intoxicated host cell’s DDR, and subsequent G2/M phase arrest and cellular distention (see Table 2) [10,51,64,65]. This is also true of S-CDT, and has been confirmed for S-CDT produced by both Typhi and NTS serotypes [8,11,12,32,33,34,42]. Interestingly, CDT-mediated DNA damage preferentially results in G2/M phase arrest [10,16,17,19,21,22,23,24,25,26,38,51,65]. However, it should be noted that cells arrested in the G2/M phase may have sustained damage prior to entering the G2 phase [51,64,66]. The majority of studies reporting G2/M phase arrest in CDT-intoxicated cell populations used DNA content to attribute cells to a defined growth phase [10,12,51,59]. However, the quantification of DNA within a given cell would not distinguish damage that occurred and was detected prior to G2 phase, versus damage occurring in G2 [51,66]. In support of this, Fedor *et al.* determined that for HeLa cells intoxicated with low doses of *E. coli* CDT, SSB were converted to DSB in the S-phase [64]. Therefore, it is likely that CDT and S-CDT DNase activity induces DNA damage regardless of the eukaryotic cell cycle phase, but the actual cell arrest is evident in the G2/M phase transition.

Following detection of DNA damage, the host cell’s DDR is activated. While the activation of DDR proteins has not been reported for S-CDT, studies of CDT-mediated intoxication for other Gram-negative bacteria have confirmed the activation of the MRN complex (a complex of Mre11, Rad50 and Nbs1) in the ataxia telangiectasia mutated (ATM) dependent DNA damage signaling pathway, as well as phosphorylation of the C-terminal serine 139 of histone H2AX (called γH2AX), which is commonly associated with DSBs [40,64,67,69,77,84,85]. In addition, single cell electrophoresis of CDT-intoxicated cells (also referred to as the “comet assay”) has demonstrated DNA fragmentation, indicating that CdtB is capable of inducing multiple lesions in the host DNA [64,85,86]. However, Fahrer *et al.* also suggested that CDT is capable of activating the ataxia telangiectasia and Rad3 related (ATR) mediated DDR signaling pathway, but at a delayed rate compared to the ATM-dependent signaling pathway [85]. Finally, the DNA damage induced by S-CDT and the CDTs of other Gram-negative bacteria causes nuclear enlargement and a distended morphology among intoxicated cells [10,11,12,14,51].

### 7.2. Apoptosis of Immune Cells and Host Immune Suppression

Apoptosis resulting from CDT-intoxication has been demonstrated for a wide range of host cell types, including immune and non-immune cell types [33,73,87,88,89,90]. Williams *et al.* demonstrated that S-CDT produced by *S*. Javiana induced apoptosis in J774A.1 macrophage cells, which also had a significant increase in expression of the pro-apoptotic *Bax* gene compared to J774.A1 cells infected with a *S*. Javiana Δ*cdtB* isogenic mutant [33]. Currently, it appears that CDT-mediated induction of apoptosis occurs primarily via the intrinsic pathway, through increased expression of *Bax* and activation of caspase 9 and subsequently caspase 3 [51,77].

### 7.3. Tumorigenesis and Carcinogenic Potential

Chronic exposure to CDT has been investigated for several Gram-negative bacterial species. Despite similar hepatic colonization levels, mice infected with CDT positive *Helicobacter hepaticus* (*H. hepaticus*) developed hepatic dysplasic nodules, while mice infected with a CDT-null mutant did not [82]. Similarly, chronic intoxication with purified *H. hepaticus* or *H. ducrecyi* CDT was associated with malignant transformations in a cell culture model [79]. Chronic inflammation is an important predisposition for cancer development [80,91,92,93]. In multiple cell culture models, administration of purified PltB (called ArtB in the study) of *S.* Typhi elicited expression of pro-inflammatory cytokines, possibly suggesting a role for S-CDT in the induction of inflammation [45]. Chronic infection with *S.* Typhi is significantly associated with gall bladder cancer, although the contribution to, or requirement for, S-CDT production has not yet been established [94,95,96]. Together, these studies implicate a potential role for CDT and S-CDT in tumorigenesis and carcinogenesis.

One of the major limitations of studying the outcomes of chronic infection with *S.* Typhi, and therefore the potential of S-CDT in tumorigenesis or carcinogenesis, has been the lack of a suitable animal model. Recently, a humanized mouse model for *S.* Typhi infection was developed [97]. Investigations into the cellular and organismal outcomes of infection with chronic exposure to S-CDT will provide important information regarding the potential for tumorigenesis or carcinogenesis associated with salmonellosis involving S-CDT positive serotypes.

### 7.4. Administration of S-CDT May Recapitulate Symptoms of Typhoid Fever

Injection of purified S-CDT recapitulated symptoms associated with the acute phase of typhoid fever, for a mouse model of infection [8]. Following systemic administration, mice injected with active S-CDT lost significantly more weight compared to control mice [8]. Furthermore, mice intoxicated with the wild type S-CDT showed a marked decrease in neutrophil counts, which is characteristic of typhoid fever in humans [8]. However, infection with NTS serotypes encoding S-CDT does not result in a typhoid-like illness [6,98,99,100]. While S-CDT may indeed contribute to typhoid fever, the widespread distribution of S-CDT among NTS, along with the marked difference in virulence between NTS serotypes and *S.* Typhi, suggest that the typhoid toxin may not solely responsible for, but may contribute to, typhoid fever. Alternatively, the discrepancy in disease severity between infections with *S.* Typhi and NTS serotypes producing an S-CDT, could reflect differences in expression of S-CDT, as alignments of toxin-encoding gene components from NTS and *S.* Typhi suggest that *cdtB*, *pltA*, and *pltB* are highly conserved [34]. Use of the recently developed humanized mouse model may provide an opportunity to further define the contribution(s) of S-CDT to human typhoid fever [97]. More specifically, it would be interesting to establish if S-CDT contributes to immune cell depletion, and if S-CDT enhances the ability of *S*. Typhi to establish a chronic infection, as has been proposed by other groups studying colonization and persistence of other CDT-producing pathogens [83].

### 7.5. Persistence and Chronic Infection

Approximately 2%–5% of *S.* Typhi infections result in chronic infection [101,102]. For *H. hepaticus*, CDT is required for colonization in a host model of infection [83]. It is possible that the cell cycle arrest and immune suppression associated with S-CDT intoxication may play an important role in the colonization and development of a chronic infection with *S*. Typhi as well.

## 8. Discussion and Future Directions

Overall, CDT has been implicated as an important virulence factor among Gram-negative bacteria, having been associated with the bacteria’s ability to colonize, survive, and persist within the host. Still, few studies have examined these effects in regards to the S-CDT produced by select *Salmonella* serotypes. Further characterization of S-CDT regulation, production, and mechanism of action will provide important information regarding the production of the toxin during different intra- and extracellular stages of infection. Furthermore, the true benefits of S-CDT to *Salmonella* during the course of an infection remain unclear. There is a clear difference in the severity of salmonellosis among NTS serotypes, with some serotypes being more frequently associated with invasive disease resulting in infections requiring hospitalization [6]. Could S-CDT play an important role in disease outcome? The long-term sequelae associated with salmonellosis are well established, yet the mechanisms by which these sequelae arise are poorly understood. For example, the well-established association between gall bladder cancer and chronic infection with *S.* Typhi may be attributable to chronic exposure to S-CDT [94,95,96]. Chronic infection with NTS is less studied, although some reports suggest that NTS may induce chronic infections in humans and in animals [103,104]. Genotoxin production by other pathogens has also been implicated in carcinogenesis in the host [73,105,106]. Further elucidation of the true long-term sequelae associated with S-CDT-mediated intoxication will provide valuable information, which may partially explain the observed differences in virulence among the NTS serotypes.

Select pathogens are differentiated based on their possessing certain virulence factors. Shiga toxin producing *E. coli* (STEC) are characterized based on the presence of *stx_1_* and *stx_2_* genes encoding shiga toxins 1 and 2, respectively [107]. In STEC infections, appropriate treatment is guided by rapid detection of the *stx* genes, as antibiotic treatment is associated with a significantly higher incidence of hemolytic uremic syndrome, and is therefore discouraged [108]. S-CDT status could influence treatment regimens, and could also serve as an epidemiological tool for comparing similar strains implicated with a common food vehicle, as is done with the *stx* genes in *E. coli* [107].

Further characterization of S-CDT has the potential to identify novel rapid detection methods for S-CDT-producing *Salmonella* in clinical settings. Characterization of this bacterial toxin may also inform the development of novel diagnostic, treatment, and prevention strategies for salmonellosis, as demonstrated previously for a variety of diseases including botulism, *Clostridium difficile* infection, and HUS resulting from infection with shiga toxin producing *E. coli* [109,110,111].

## 9. Conclusions

Overall, the implications of S-CDT in the context of salmonellosis present a unique and intriguing challenge. Multiple CDT-producing pathogens have been linked to an increased incidence of cancer among chronically infected individuals [80,95,96]. The public health implications of S-CDT production by NTS should be considered, as the recent discovery of the widespread nature of the toxin among NTS suggests that, at least in the US, many individuals may be exposed to S-CDT. Future investigations relating S-CDT’s role in pathogenesis, as well as implications for the long-term sequelae attributable to S-CDT-mediated intoxication will be beneficial in assessing the contributions of S-CDT to salmonellosis in both humans and animals.

## Figures and Tables

**Table 1:** Salmonella cytolethal distending toxin (S-CDT) status of select *Salmonella enterica* subspecies *enterica* serotypes.

Serotype	S-CDT Status ^1^	References
9,12:I,v:-	−	[9]
Agbeni	+	[13]
Agona	−	[13]
Anatum	−	[13]
Arechavaleta	+	[13]
Bareilly	−	[13]
Barranquilla	+	[13]
Berta	−	[13]
Braenderup	−	[13]
Brandenburg	+	[13]
Bredeney	+	[9]
Choleraesuis	−	[9,13]
Corvallis	+	[13]
Cotham	+	[13]
Cubana	+	[13]
Dublin	−	[9,13]
Enteritidis	−	[9,13]
Freetown	+	[13]
Gaminara	+	[13]
Georgia	+	[13]
Give	+	[13]
Glostrup	+	[13]
Hadar	−	[9,13]
Hartford	−	[13]
Heidelberg	−	[9,13]
4,[5],12:i:-	−	[13]
Indiana	+	[13]
Infantis	−	[13]
Inverness	+	[13]
Javiana	+	[13,32]
Johannesburg	+	[13]
Kiambu	+	[13]
Kintambo	+	[13]
Kisarawe	+	[13]
Luciana	+	[13]
Miami	+	[13]
Minnesota	+	[13]
Mississippi	±	[13]
Montevideo	+	[9,13,36]
Muenchen	−	[13]
Muenster	+	[13]
Newport	−	[9,13]
Oranienburg	+	[13]
Overschie	+	[13]
Panama	+	[13]
Paratyphi A	+	[13]
Pomona	+	[13]
Poona	+	[13]
Reading	+	[13]
Rubislaw	+	[13]
Sandiego	+	[13]
Schwarzengrund	+	[9,13]
Telelkebir	+	[13]
Thompson	−	[13]
Typhi	+	[12,29]
Typhimurium	−	[9,13]
Urbana	+	[13]
Virchow	−	[9,13]
Wandsworth	+	[13]

^1^ Status based on the presence of all S-CDT encoding genes (*pltA, pltB*, and *cdtB*) as determined by PCR-based amplification; “+” denotes all genes are present; “−” denotes one or more genes were not detected; “±” denotes some isolates within the serotype are positive, but others are negative (unpublished data).

**Table 2:** Pathogenic outcomes attributed to intoxication with Salmonella cytolethal distending toxin and other Gram-negative bacteria producing cytolethal distending toxins.

Pathogenic Outcome of CDT-Mediated Intoxication	Bacterial Species ^1^	References
**Cellular Outcomes**		
G2/M Phase arrest	*A. actinomycetemcomitans*	[12,14,15,17,19,22,24,27,28,34,67]
	*C. jejuni*	
	*E. coli*	
	*Haemophilus* spp.	
	*Helicobacter* spp.	
	*P. alcalifaciens*	
	*Shigella* spp.	
	*Salmonella* (Typhi and NTS)	
Activation of host cell DNA damage response	*A. actinomycetemcomitans*	[26,28,40,64,68,69,70,71]
	*C. jejuni*	
	*E. coli*	
	*Haemophilus* spp.	
	*H. ducreyi*	
	*H. hepaticus*	
	*P. alcalifaciens*	
	*S.* Typhi	
Induction of autophagy	NTS	[33]
Induction of apoptosis	*A. actinomycetemcomitans*	[33,72,73,74,75,76,77,78]
	*C. jejuni*	
	*E. coli*	
	*H. ducreyi*	
	*Helicobacter* spp.	
	*P. alcalifaciens*	
	NTS	
**Host Outcomes**		
Tumorigenesis and neoplastic lesions	*H. cinaedi*	[79,80,81,82]
	*H. hepaticus*	
	*H. ducreyi*	
Typhoid-like illness	*S.* Typhi	[8]
Chronic infection	*H. hepaticus*	[83]

^1^ Pathogenic outcome reported for CDT produced by given bacterial species. NTS refers to “nontyphoidal *Salmonella*”.

## Abbreviations

The following abbreviations are used in this manuscript:

S-CDT	Salmonella Cytolethal Distending Toxin
CDT	Cytolethal Distending Toxin
NTS	nontyphoidal *Salmonella*
DDR	DNA Damage Response
SCV	Salmonella Containing Vacuole
